# Ascending aortic length across a large population presenting to the emergency room, a retrospective cross-sectional study

**DOI:** 10.1016/j.ijcha.2026.101874

**Published:** 2026-01-18

**Authors:** Thomas Saliba, Gabriella Giandotti Gomar, Olivier Cappeliez, Yasser Alemán-Gómez, Guillaume Fahrni, David Rotzinger

**Affiliations:** a Radiology Department, CHUV, Lausanne, Switzerland; b Free University of Brussels (VUB), Brussels, Belgium; c Thoracic surgery department, CHUV, Lausanne, Switzerland; d Braine L’Alleud Hospital (CHIREC), Braine L’Alleud, Belgium

**Keywords:** Aortic length, Ascending aorta, type A dissection, Table, CT-scan

## Abstract

**Background:**

Aortic dissection often occurs at diameters below surgical thresholds, underscoring the need for better predictive markers. Ascending aortic length has emerged as a potential morphologic risk factor, but normal population data are limited. This study aimed to establish normative aortic length values by age and sex and develop a tool to predict dissection risk.

**Methods:**

We retrospectively analyzed 1030 (986 without and 44 with type A dissection) emergency room patients (from 1,445 screened) who underwent ECG-gated thoracic CT angiography between 2019 and 2025, excluding those with prior aortic surgery or disease. Ascending aortic length, from the sinotubular junction to the brachiocephalic trunk, was measured using semi-automated centerline tools. Logistic and LASSO regression models estimated type A dissection probability based on aortic length, age, height, and sex.

**Results:**

Mean ascending aortic length was 70.7 ± 11.6 mm in men and 64.1 ± 11.4 mm in women. Patients with acute type A dissection (n = 44) had significantly longer aortas (men: 93.9 ± 20.5 mm; women: 90.0 ± 18.5 mm; p < 0.001). Aortic length was the strongest independent predictor (OR = 1.13, 95 % CI 1.10–1.17, p < 0.001). A reduced model including only aortic length showed excellent discrimination (AUC = 0.871; sensitivity = 0.773; specificity = 0.867; PPV = 0.206; NPV = 0.988).

**Conclusion:**

Ascending aortic length increases with age and is markedly greater in patients with acute type A dissection. We provide normative reference tables by age and sex and a logistic model for individualized risk estimation of dissection at the time of the exam.

## 1 Introduction

Aortic dissections, which can be classified as A or B-type or Non-A/Non-B depending on their location relative to the aortic arch, are rare but can be catastrophic if not treated rapidly [1]. Type A aortic dissections have a hospital mortality exceeding 50 % for patients without surgical repair, and an incidence of 2–10 cases per 100 000 people [2], [3]. Though current guidelines for thoracic aortic aneurysm repair are based on diameter, recent studies have shown that 97 % of dissections affect patients outside of the guidelines for prophylactic aortic repair [1], [2], [3]. Furthermore, 68 % of patients still would not reach the 55 mm diameter threshold required post-dissection [3].

Emerging evidence suggests that ascending aortic length is a risk factor for aortic dissections, with a cut-off of 11 cm being suggested by Wu et al following an apparent increase in dissections in patients with ascending aortas ranging from 11.5 to 12 cm in length [4]. One study found that patients who had suffered an A-type dissection had aortas that were, on average, 16 mm longer pre-dissection than patients without aortic disease, and 12 mm longer than patients with aortic aneurysms, with other studies showing similar results [4], [5].

Increasing length of the ascending thoracic aorta has been associated with ageing [6]. This is likely explained by the greater elastin content in the aortic arch and proximal descending portion, making these areas the most vulnerable to remodelling [6]. Furthermore, studies describe an average lengthening of the ascending aorta of 59 mm for males and 66 mm for females from 20 to 80 years of age [6].

The pathophysiology linking ascending aortic length and dissection remains debated. It is possible that the lengthening of the aorta is a consequence of the breaking of elastin fibres, which is accompanied by a decreased vascular compliance [7]. Some authors also hypothesised that, based on the transverse tear which is found upon intimal inspection that would suggest stress in the longitudinal axis, that aortic lengthening over time could play an important role in dissection [3]. Although the exact pathophysiology remains unknown, evidence points to a link between aortic dissection and aortic length.

Although the fact that aortic length increases with age has been previously reported, these studies were relatively limited in size, numbering fewer than 200 patients. In a similar study, Zafar et al created a risk table for aortic dissection based on aortic diameter normalised for patient height, though as previously stated aortic diameter remains a poor predictor of dissection [8]. Therefore, the objective of our study was to conduct a cross-sectional retrospective study in order to produce average expected proximal aortic lengths for a given population according to age and size.

## 2 Methods

We performed a retrospective cross-sectional study of aortic length using patients whose CT-scans included the acquisition of an ECG-gated thoracic CT angiogram performed in the emergency department between January 1, 2019, and January 21, 2025. The database search identified 1,445 patients.

Inclusion criteria for the non-dissected cohort were age ≥18 years, absence of prior aortic surgery, and no acute aortic pathology. Patients meeting these criteria but presenting with acute type A dissection were included in the dissection group.

Ascending aortic aneurysms were not a cause for exclusion due to their high prevalence in the general population, with around 13 % expected to have an aortic diameter of >36 mm [9]. Therefore this was not used as an exclusion factor.

Exclusion criteria were CT-scans that were mislabelled in the PACS (eg: abdominal or head CT) and thus did not have an ECG-gated aortic angiography CT-scan, CT-scans that were subject to motion artefacts that made them uninterpretable, exams for which the vessel measurement software was not able to correctly segment the aorta, patients with previous aortic surgery, missing data and refusal of data use for research. Step artefacts due to the acquisition mode were not considered as motion artefacts.

All of the exams were acquired on a GE Healthcare (Boston, USA) Revolution series scanner using Accupaque 350 (GE Healthcare, Oslo, Norway) contrast agent. All exams that were labelled as ECG-gated CT angiograms that included the chest were deemed admissible. Therefore, both CT scans limited to the chest as well as thoracoabdominal and whole-body CT-scans were considered eligible if they included an ECG-gated thoracic aortic angiogram. The part of the cardiac cycle used for the reconstruction was not recorded.

As with similar cross-sectional studies, we did not control for confounding factors or underlying diseases such as blood pressure, diabetic condition, smoking history, and family history, reasoning that as a cross-sectional survey of the population it would provide a representative sample of patients [8].

We also did not control for the indication for which the scan was performed, electing to include any patient who underwent an ECG-gated CT angiogram at the emergency department.

**Fig. 1:**
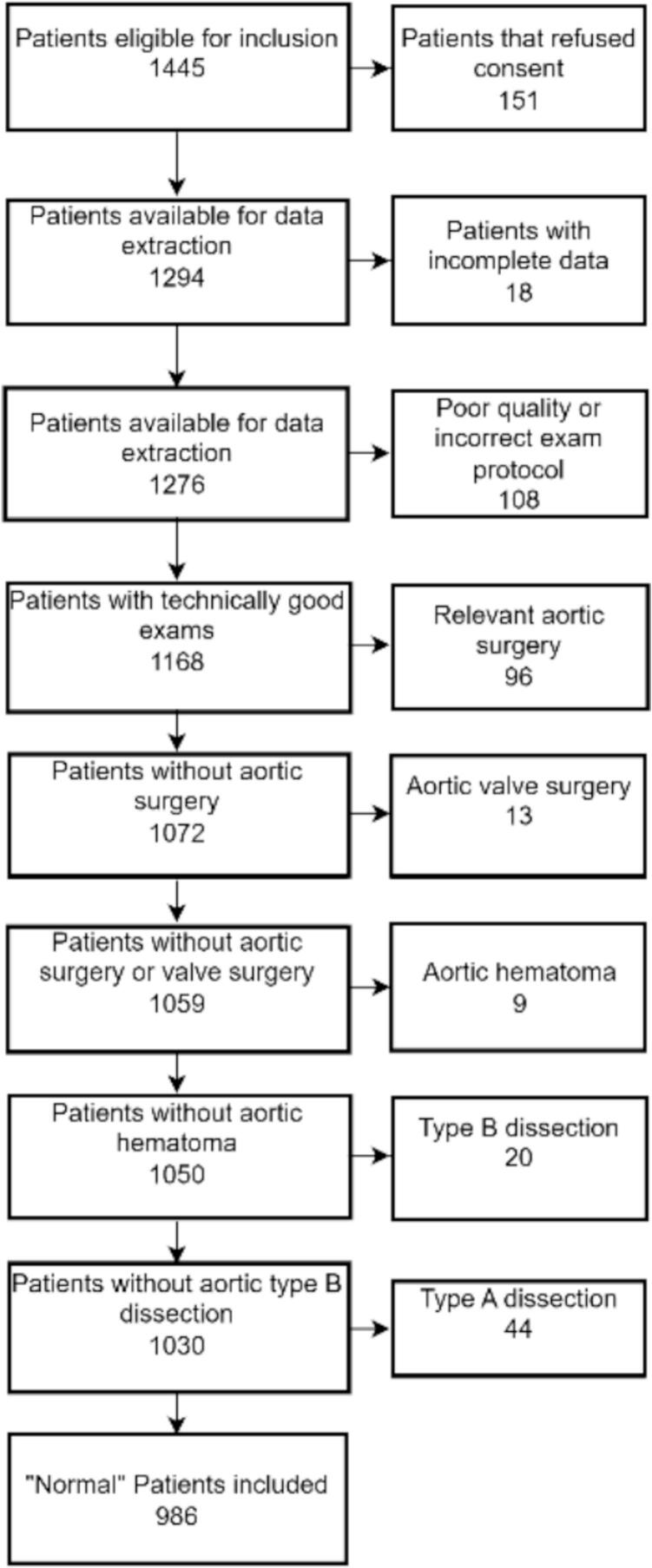
Patient inclusion flowchart.

The patient inclusion flowchart is depicted in Fig. 1.

**Fig. 2:**
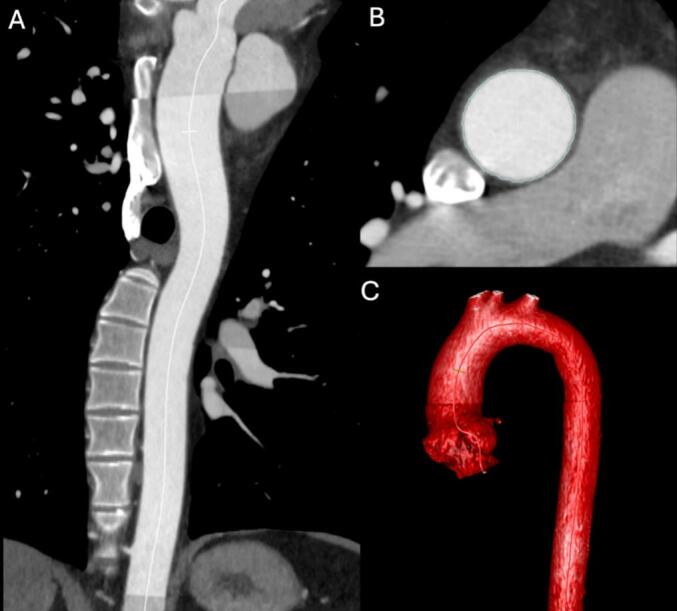
Post-processing of a thoracic aortic computed tomography angiography (CTA). (A, B) Centerline reformations of the thoracic aorta in mediastinal window settings, displaying orthogonal cross-sectional planes along the aortic lumen. Panel A shows the long-axis view, while Panel B shows the corresponding short-axis view perpendicular to the aortic centerline at the level of the ascending thoracic aorta. (C) Three-dimensional (3D) external volume-rendered (VR) reconstruction of the thoracic aorta.

The measurements of the aortic length were performed using the Philips PACS (Picture Archiving and Communication System) using in-built semi-automated vessel post-processing tools using a centerline measurement to ensure geometric accuracy. The measurements were performed by a single radiologist with 4 years of experience. The ascending aorta was measured from the sinotubular junction to the origin of the brachiocephalic trunk, in accordance with the most commonly used definition (Fig. 2).[6], [7], [10] Patient age, sex, and height were obtained from self-reported data entered at the time of imaging.

Ethical approval was obtained from the Ethics committee of the canton of Vaud for human research (CER-VD) of the Vaud canton in Switzerland under the identification number 2025-00512.

Statistical analysis was conducted using RStudio (version 2024.04.2 Build 764) with statistical significance set at p < 0.05.

Expected aortic lengths by age and sex were tabulated. Logistic regression was used to model dissection presence as the dependent variable, with aortic length, age, height, and sex as predictors. Least Absolute Shrinkage and Selection Operator (LASSO) regression with 10-fold cross-validation was applied to avoid overfitting, yielding two predictive models.

The logistic model estimated the probability of aortic dissection at a single time-point. We chose this design in the absence of available longitudinal data, which is challenging to obtain because patients often present with acute dissections without a documented imaging history over a longitudinal period.

Odds ratios (ORs) with 95 % confidence intervals (CIs) were derived from the regression coefficients. Model performance was evaluated using the area under the receiver operating characteristic curve (AUC), Akaike information criterion (AIC), and Brier score, with internal validation by cross-validation. P-values < 0.05 were considered significant.

## 3 Results

After the exclusion of patients with scans that were not interpretable due to motion artefacts, 1030 patients remained (61 % male; mean age 64 years in males and 66 years in females). The mean aortic length was 70.7 mm (±11.6 mm) for male patients and 64.1 mm (±11.4 mm) for female patients (Table 1). Of these patients, 986 (95 %) had not experienced an aortic dissection and 44 (5 %) had experienced an acute type A aortic dissection. The average aortic length for patients who had experienced a type A dissection was 93.9 mm (±20.5 mm) and 90.0 mm (±18.5 mm) for males and females, respectively (Table 1).

**Table 1:** Summary of the main characteristics of the cohorts.

	Male − Non-Dissected	Female − Non-Dissected	Male − Dissected	Female − Dissected	Missing Data	Median [IQR]	95 % Ref Range	Normality	Test Statistic	Effect Size	P-value
	(N = 604)	(N = 382)	(N = 30)	(N = 14)							
Age											
Mean (SD)	64.1 (16.1)	66.3 (16.6)	60.9 (14.0)	74.0 (14.5)	0 (0.0 %)	MND: 67.0 [55.0–77.0] FND:69.0 [56.2–79.0] MD: 62.0 [52.2–69.5] FD: 78.5 [67.8–83.8]	MND: 27.0–88.0 FND: 31.5–92.0 MD: 34.5–87.5 FD: 44.9–91.7	M: Non-normal | F: Non-normal	M: W = 10653.5 | F: W = 1917.5	M: d = 0.20 (small) | F: d = 0.47 (small)	M: 0.104 | F: 0.072
Median [Min, Max]	67.0 [18.0, 98.0]	69.0 [18.0, 97.0]	62.0 [28.0, 94.0]	78.5 [39.0, 93.0]							
Height											
Mean (SD)	176 (7.35)	163 (7.21)	177 (4.64)	164 (6.03)	0 (0.0 %)	MND: 175.0 [170.0–180.0] FND: 165.0 [160.0–168.0] MD: 180.0 [174.2–180.0] FD: 166.5 [160.8–169.5]	MND: 160.0–190.0 FND: 150.0–178.0 MD: 170.0–185.0 FD: 152.3–170.0	M: Non-normal | F: Non-normal	M: W = 8046.0 | F: W = 2330.5	M: d = 0.18 (negligible) | F: d = 0.15 (negligible)	M: 0.297 | F: 0.412
Median [Min, Max]	175 [150,198]	165 [131,185]	180 [170,185]	167 [151,170]							
Ascending Aortic Length											
Mean (SD)	71 (11.6)	64 (11.4)	94 (20.5)	90.0 (18.5)	0 (0.0 %)	MND: 70.0 [63.0–78.0] FND: 64.0 [57.0–70.8] MD: 86.0 [81.2–110.5] FD: 90.0 [75.2–97.2]	MND: 49.0–95.0 FND: 42.5–89.0 MD: 69.7–137.0 FD: 63.9–122.4	M: Non-normal | F: Non-normal	M: W = 2670.0 | F: W = 556.5	M: d = 1.92 (large) | F: d = 2.21 (large)	M: <0.001 | F: <0.001
Median [Min, Max]	70 [42.0, 111]	64 [21.0, 103]	86 [69.0, 137]	90 [61.0, 125]							

MND: Male non-dissected, FND: Female non-dissected, MD: Male dissected, FD: Female dissected.

To verify that our results were not due to differences in the characteristics within our population, we performed an analysis whilst matching patients with and without dissections for age, height and sex. The average aortic length for those who had not experienced a dissection was 65.3 mm (±8.24 mm) and 66.7 mm (±11.7 mm) for males and females, respectively, and 93.9 mm (±20.5 mm) and 90.0 mm (±18.5 mm) for those who had undergo a type A dissection (Table 2). The differences were found to be significant for both males and females (p < 0.001).

**Table 2:** Patients without and without dissection matched for age, sex and height.

	Male − Non-Dissected	Female − Non-Dissected	Male − Dissected	Female − Dissected	Missing Data	Median [IQR]	95 % Ref Range	Normality	Test Statistic	Effect Size	P-value
	(N = 30)	(N = 14)	(N = 30)	(N = 14)							
Age											
Mean (SD)	60.9 (13.9)	74.1 (14.1)	60.9 (14.0)	74.0 (14.5)	0 (0.0 %)	MND: 62.0 [53.0–68.8] FND: 78.5 [67.2–84.0] MD: 62.0 [52.2–69.5] FD: 78.5 [67.8–83.8]	MND: 35.5–88.0 FND: 46.5–92.0 MD: 34.5–87.5 FD: 44.9–91.7	M: Normal | F: Normal	M: t = 0.02 | F: t = 0.03	M: d = 0.00 (negligible) | F: d = 0.01 (negligible)	M: 0.985 | F: 0.979
Median [Min, Max]	62 [29.0, 96.0]	79 [41.0, 94.0]	62 [28.0, 94.0]	79 [39.0, 93.0]							
Height											
Mean (SD)	177 (4.59)	165 (5.85)	177 (4.64)	164 (6.03)	0 (0.0 %)	MND: 180.0 [175.0–180.0] FND: 166.5 [161.0–169.5] MD: 180.0 [174.2–180.0] FD: 166.5 [160.8–169.5]	MND: 170.0–184.3 FND: 153.0–170.0 MD: 170.0–185.0 FD: 152.3–170.0	M: Non-normal | F: Non-normal	M: W = 459.0 | F: W = 100.5	M: d = 0.01 (negligible) | F: d = 0.04 (negligible)	M: 0.893 | F: 0.925
Median [Min, Max]	180 [170,185]	167 [152,170]	180 [170,185]	167 [151,170]							
Ascending Aortic Length											
Mean (SD)	65 (8.24)	67 (11.7)	94 (20.5)	90 (18.5)	0 (0.0 %)	MND: 64.5 [59.5–71.8] FND: 66.0 [59.2–71.5] MD: 86.0 [81.2–110.5] FD: 90.0 [75.2–97.2]	MND: 51.4–77.8 FND: 49.3–89.4 MD: 69.7–137.0 FD: 63.9–122.4	M: Non-normal | F: Normal	M: W = 54.0 | F: t = -3.98	M: d = 1.83 (large) | F: d = 1.50 (large)	M: <0.001 | F: <0.001

MND: Male non-dissected, FND: Female non-dissected, MD: Male dissected, FD: Female dissected.

**Fig. 3:**
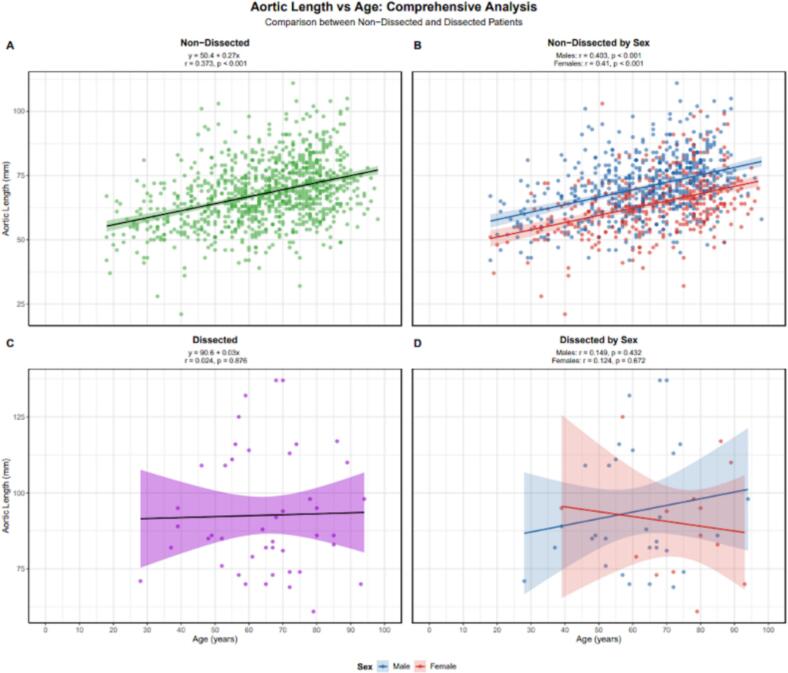
Aortic length versus age comparison for patients without aortic dissections with both sexes combined (top left) and separated (top right). Aortic length versus age comparison for patients with type A dissections with both sexes combined (bottom left) and separated (bottom right).

**Fig. 4:**
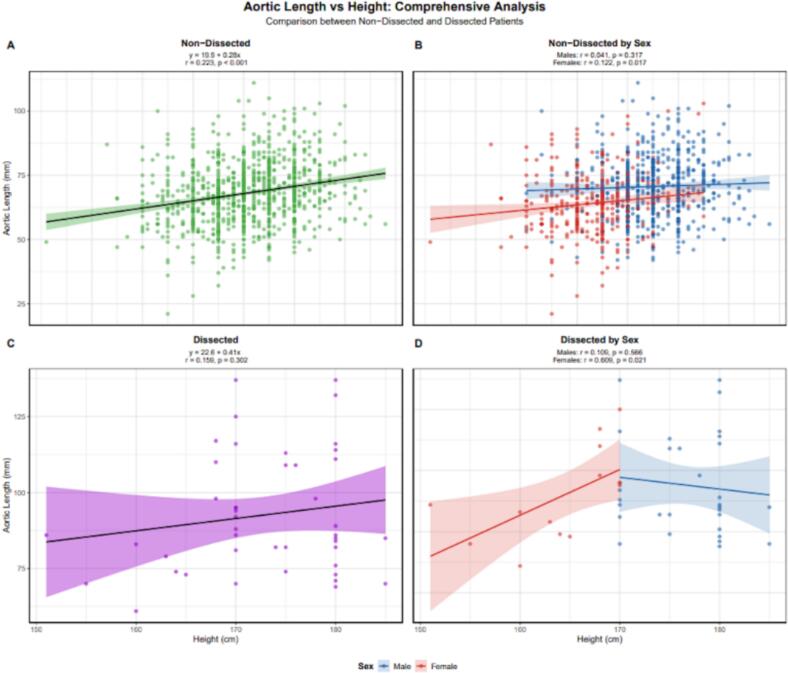
Aortic length versus patient height comparison for patients without aortic dissections with both sexes combined (top left) and separated (top right). Aortic length versus age comparison for patients with type A dissections with both sexes combined (bottom left) and separated (bottom right).

We produced a nomogram table for both male (Table 3) and female (Table 4) patients, showing the expected aortic length based on age. Aortic length appeared to be a better predictor than height, as indicated by higher R^2^ values. These were 0.373 vs. 0.233 for the overall population, 0.403 vs. 0.041 for males, and 0.41 vs. 0.122 for females (Fig. 3, Fig. 4).

**Table 3:** Table of the average aortic lengths for males according to age.

Age Group	Sex	n	Mean ± SD (mm)	Median [IQR] (mm)	95 % Ref Range (mm)
10–19	Male	**2**	54.5 ± 17.7	54.5 [48.2–60.8]	42.6–66.4
20–29	Male	**24**	58.4 ± 8.8	57.5 [53.5–62.5]	44.1–76.4
30–39	Male	**27**	60.2 ± 9.4	58.0 [53.0–67.0]	45.6–76.5
40–49	Male	**58**	67.2 ± 11.9	66.0 [58.0–73.5]	51.4–93.6
50–59	Male	**97**	68.3 ± 10.1	69.0 [62.0–74.0]	47.2–86.8
60–69	Male	**137**	69.9 ± 9.8	69.0 [63.0–76.0]	52.4–90.6
70–79	Male	**154**	74.5 ± 11.1	74.5 [67.0–80.0]	52.8–99.0
80–89	Male	**98**	76.3 ± 11.2	76.0 [70.0–83.8]	55.3–100.6
90+	Male	**7**	70.4 ± 6.6	74.0 [67.5–74.5]	59.3–76.7

**Table 4:** Table of the average aortic lengths for females according to age.

Age Group	Sex	n	Mean ± SD (mm)	Median [IQR] (mm)	95 % Ref Range (mm)
10–19	Female	**2**	44.0 ± 9.9	44.0 [40.5–47.5]	37.4–50.6
20–29	Female	**4**	48.2 ± 5.7	48.5 [46.2–50.5]	41.5–54.6
30–39	Female	**23**	56.2 ± 9.1	55.0 [52.5–62.0]	40.1–72.0
40–49	Female	**38**	57.3 ± 10.4	57.5 [52.2–64.0]	34.9–73.0
50–59	Female	**56**	62.7 ± 10.2	62.5 [56.0–67.2]	47.1–83.0
60–69	Female	**76**	62.4 ± 10.6	62.0 [54.8–68.2]	44.6–83.4
70–79	Female	**90**	67.8 ± 11.0	66.0 [60.5–74.8]	48.4–90.5
80–89	Female	**76**	68.9 ± 11.3	67.5 [61.0–74.2]	50.8–95.5
90+	Female	**17**	67.3 ± 5.9	67.0 [64.0–72.0]	57.0–76.4

We subsequently produced predictive models to calculate the risk of occurrence of type-A aortic dissection based on age and length, using logistic regressions.

The probability of aortic dissection at the time of evaluation, accounting for aortic length, age and height, was expressed using the following logistic regression equations:

$$\rho =\frac{1}{1+e^{-(-4.6673+0.1235(aortic\;length)-0.0379(age)-0.0326(height))}}$$

where p represents the probability of aortic dissection at the time of the examination.

The reduced model (without accounting for height) was expressed as:

$$\rho =\frac{1}{1+e^{-(-4.6673+0.1235(aortic\;length))}}$$

where p represents the probability of aortic dissection at the time of the examination.

We conducted a multivariate analysis of the study cohort (n = 1,030; 44 with dissection (4.3 %)) showing that aortic length was the strongest independent predictor of dissection. In the reduced logistic regression model, longer aortic length was associated with a significantly increased odds of dissection (OR = 1.13, 95 % CI: 1.10–1.17, *p* < 0.001). Age was inversely associated with dissection risk (OR = 0.96, 95 % CI: 0.94–0.99, *p* = 0.005), whereas height showed no statistically significant effect (OR = 0.97, 95 % CI: 0.93–1.01, *p* = 0.14).

The reduced model demonstrated excellent discrimination with an AUC of 0.871 (95 % CI: 0.814–0.929) and good overall calibration. Model fit was comparable to the full model (AIC 251.7 vs. 253.0), indicating that the reduced model achieved equivalent predictive accuracy with fewer variables.

At the optimal cutoff (Youden Index = 0.64), the model yielded a sensitivity of 0.773, specificity of 0.867, positive predictive value of 0.206, and negative predictive value of 0.988.

**Fig. 5:**
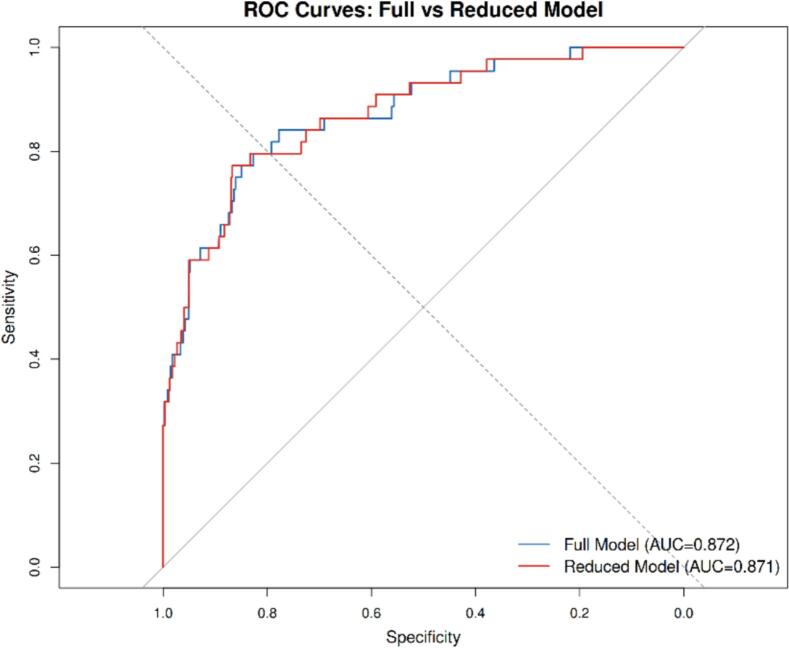
Receiver operating characteristic graph for the full model and the reduced model for predicting aortic type A aortic dissection.

The ROC for both models are shown in Figure 5.

## 4 Discussion

In this study, we established average ascending aortic lengths across populations stratified by sex, age and height, and used this data to create normative tables of expected aortic length. We then compared the results with those from patients in our emergency department cohort who experienced type A aortic dissection to assess for statistically significant differences between groups. Our findings revealed such differences, corroborating prior findings that aortic elongation is an important but under-recognized risk factor.

Aneurysms of the ascending aorta are a well-established risk factor for aortic dissection, with thresholds for surgery based on diameter being commonly used [1], [11]. However, it is well known that aortic dissection can occur at diameters considered well below the threshold for preemptive surgical intervention in what is referred to as the “aortic size paradox” [12], [13]. This has led to the search for alternative ways of determining aortic dissection risk, with ascending aortic length emerging as a possible factor.

The length of the ascending aorta was previously investigated by a number of different groups [3], [4], [5], [7], [10], [14], [15], [16]. However, these previous studies often varied in their methodology and thus produced varying estimations for ascending aortic lengths.

Krüger et al attempted to measure the ascending aortic length in 2016 using a method based on frontal and sagittal reconstructions of healthy and dissected aortas [5].

Members of the same group then conducted a study on the ascending aortic length and attempted to derive a prediction for dissection risk [10]. However, their sample was not solely composed of ECG-gated exams, which could cause imprecise measurement due to motion artifacts. Furthermore, they defined the ascending aorta as beginning from the aortic valve to the brachiocephalic trunk, instead of using the more commonly accepted definition that describes the sinotubular junction as the proximal point. They concluded that patients with aortic diameters of 45–54 mm, alongside elongations over 120 mm were a high-risk subgroup for whom prophylactic surgery should be discussed.

Korpela et al conducted a similar study, reporting significantly longer aortic lengths in patients who had suffered type A dissections compared to those with non-dissected aortas (110 ± 15 mm vs 97 ± 11 mm) [14]. However, they also used the annulus to brachiocephalic trunk as their definition of ascending aorta [14]. They found that men had significantly longer ascending aortas than women, measuring 11 mm more, with this figure rising to a difference of 15 mm in dissected aortas [14].

Our findings that age has a positive correlation with ascending aortic length is accordance with previous findings, further reinforcing that this is the driving factor increasing aortic length [17]. However, we also found that age had a slight negative correlation with the risk of type A aortic dissection. Though this is counter-intuitive, it may be explained by the lower incidence in older populations, as has been shown by large scale studies that demonstrate a peak incidence at around 50 to 54 years old [18]. However, it may also be explained by a bias induced by the relatively few patients suffering from dissections who were included in the study as it goes against the findings of other, larger, studies [19].

Our emergency department cohort had an average ascending aortic length of 70.7 mm in males and 64. 1 mm in females, similar to those found by many studies that also used the same methodology of measuring from the sinotubular junction to the brachiocephalic trunk [3], [5], [10]. However, it is below the average ascending aortic length found by others, who suggest average figures of 97 mm (using an annulus to brachiocephalic trunk measurement) [14]. Another study found that the ascending aortic length before dissection was 104.8 mm, whilst being slightly lower at 95.4 mm in patients who thoracic aortic aneurysms before dissection (using a sino-tubular junction to brachiocephalic trunk measurement) [7].

Another study found an average ascending aortic length 112 mm in patients without acute aortic dissection and 111 mm in those who suffered aortic dissections (using an annulus to brachiocephalic trunk measurement) [20].

These results, pointing to similar sizes in both groups, is surprising and constitutes an outlier in the context of the wider literature [20]. However, in their study, the authors did not comment on this fact. Instead, they focused on the limited change between the pre-dissection and post-dissection measurements in some patients of their cohort [20]. They argued that the lack of change in aortic length post-dissection provides a cleaner way of setting thresholds for intervention than the aortic diameter, which changes after dissection [20].

We suspect that the differences between our results and those of previous studies stem from differing inclusion criteria. Our less stringent approach, analyzing any CTA performed in the emergency department, likely provided a better sampling of the general population, rather than patients with pre-existing cardiovascular risks, as was the case in some studies [20]. Furthermore, the differences in measurement methodology likely explain the larger differences between aortic lengths. As one would expect, those that measured from the annulus to the brachiocephalic trunk recorded longer lengths than those that used the sinotubular junction to the brachiocephalic trunk measurement as we did.

In order to provide a comparison of the different methods of aortic measurements and the reported aortic lengths, we have summarized them in Table 5.

**Table 5:** 

Study	Measurements	Average age (years)	Control length (mm)	Pathological aneurysm length (mm)	Pathological dissection length (mm)	Pathological pre-dissection length (mm)
Korpela (2023)	Annulus to brachiocephalic trunk	65 ± 16 (healthy) 69 ± 11 (dissection)	97 ± 11		110 ± 15 mm	
Li (2024)	Sinotubular junction to brachiocephalic trunk	53.99 ± 11.45 (aneurysm), 53.37 ± 12.17 (dissection)		95.4 ± 16.7		104.8 ± 14.8
Sun (2022)	Annulus to brachiocephalic trunk	65 ± 15	81			
Heuts (2018)	Sinotubular junction to brachiocephalic trunk	65 ± 13 (healthy) 63 ± 11 (dissection)	68.9 ± 7.2		78.6 ± 8.8	
Eliathamby (2021)	Annulus to brachiocephalic trunk	Median 62 (healthy), 65 (aneursym), 60 (dissection)	Median 83.2; iqr, 74.5–90.7		Median, 109.7; iqr, 101.0–115.1	Median, 104.2; iqr, 96.0–109.3
Kruger (2018)	Sinotubular junction to brachiocephalic trunk	Median 64 (healthy), 69,5 (aneursym), 65,2 (pre-dissection), 66,3 (dissection)	Median 69	Median 98	Median 92	Median 87
Kruger frontal plane (2016)	Sinotubular junction to brachiocephalic trunk	Median 63 (healthy), 65 (dissection)	Median 71; iqr, 64–80		Median 92; iqr, 84–102	
Kruger sagittal plane (2016)	Sinotubular junction to brachiocephalic trunk	Median 63 (healthy), 65 (dissection)	Median 66; iqr, 59–75		Median 87; iqr, 76–96	
Wu (2019)	Annulus to brachiocephalic trunk	65.8 ± 13.6	112 ± 13		111 ± 15	114 ± 14
Sugawara (2008)	Sinus of valsalva to the top of the aortic arch	54 ± 15 (japan) 43 ± 18 (usa)	75 ± 20 (japan) 62 ± 16 (usa)			
Our study	Sinotubular junction to brachiocephalic trunk	65.0 ± 16.0 (non-dissected), 64.9 ± 15.6 (dissection)	69 ± 13.5		69.6 ± 13.5	

Although the number of patients included in our study who had type A aortic dissections was relatively small, making up only 4.7 % and 3.5 % of male and female subjects, respectively, our results are in agreement with previous findings that aortic lengths are longer in patients who suffer type A dissections [4], [5]. This suggests that aortic length may be a relevant predictor for the risk of aortic dissections and that it is a parameter that should be taken into account.

Attempts have been made to designate cut-off points for the risk of aortic dissection based of ascending aortic length in the past. One group attempted to estimate risk based on ascending aortic diameter and length combined with respect to the patient’s height [20]. However, this method may fall short due to using the patient’s height rather than their age. This is because it is known that the ascending aortic length is more closely related to a patient’s age rather than their stature, which we have once again shown in our study [3], [6], [16]. They also proposed two “hinge points” at which aortic events may occur between 115 to 120 mm and 125 to 130 mm, whilst noting a large increase of 32 % probability of aortic events in a person with an aorta ≥ 130 mm, compared to < 70 mm [20]. This led them to propose 110 mm as a reasonable cutoff, suggesting that patients with ascending aortic lengths longer than that should be considered for surgical repair [20]. However, it should be noted that these results, and the subsequent recommendations, are based on the non-standard annulus to brachiocephalic trunk definition of the ascending aorta and thus can be misleading if one is not aware of this when making predictions.

We provide a table of normative values for aortic length according to a patient’s sex and age. It should be noted data does not have longitudinal follow-ups of patients who have experienced dissections which means that predictions for type A aortic dissections cannot be made beyond the immediate time at which the exam was acquired. However, it does allow for the creation of a Z-score $z=\frac{\left(measuredaorticlength)-(meanaorticlengthforgivenageandsex\right)}{\mathrm{standarddeviationoftheaorticlengthforagivenageandsex}}$, which can be used to judge which patients have aortic lengths that are above average for their sex and age. With further clinical validation, this may eventually be used to prompt closer follow-ups or pre-emptive surgical intervention in patients with aortic lengths above what would be expected.

This study also attempts to create a predictive model for the likelihood of dissection based on aortic length at a certain point in time. Our reduced model had a sensitivity of 0.773, specificity of 0.867, positive predictive value of 0.206, and negative predictive value of 0.988, allowing for a rapid estimation of the risk of dissection based solely on aortic length. Though this cannot be used to extrapolate the risk of future dissection over extended periods of time, it may prove useful to alert physicians that urgent action is needed to pre-emptively take care of patients that are at high risk of dissection.

However, the positive predictive value of our model is relatively poor. Though this may be due to it being under-powered because of our sample having few cases of patients with dissections. It may also reflect the inherent challenge in predicting the occurrence of acute aortic events as other models attempting to determine aortic dissection risk have low positive predictive values and high negative predictive values as our model does [21], [22].

Our study has several limitations. Firstly, we did not control for co-morbidities, such as hypertension, hypercholesterolemia, cardiac pathologies, or medications that the patients were taking. Although this may have enhanced our ability to examine our data in more detail, as we included patients presenting to the emergency department for any ailment resulting in a thoracic CT angiography, we believed that this would be a representative sample of the general population. Secondly, our study is monocentric, though it does benefit from being performed in a cosmopolitan hospital with patients from a wide range of backgrounds. Thirdly, the measurements were conducted by a single radiologist. However, due to measurements being semi-automated and the results closely matching what has been previously described it is likely that they are accurate. Fourthly, we did not follow up the patients over time, so we are unable to know how many of the patients without dissections at the time of the examination went on to develop dissections. This means that our predictive model for type A aortic dissections is only valid for patients at the time of the exam. Fifthly, although we included patients with aortic aneurysms as part of the normal patients due to its high prevalence amongst patients who do not have dissections, it is possible that this may introduce a bias. A sixth limitation is that our study population may be inherently biased due to the patients having been sourced from the emergency department and possibly having pathologies other than aortic pathologies. Furthermore, it is possible that our study is under powered due to the limited number of patients with aortic dissections included in the analysis.

Nevertheless, our results provide a valuable foundation for future longitudinal validation and integration of morphologic and clinical predictors by providing aortic length measurements of the largest cohort to date.

Further research is needed to validate our model for predicting type A aortic dissection risk and determine whether it can forecast dissections over longer periods.

## 5 Conclusion

In this large, retrospective, cross-sectional study, we provide normative data on ascending aortic length of cross-section of the population presenting at an emergency department, stratified by age and sex.

We confirm that there is a significant difference in ascending aortic lengths between populations that have suffered a type A dissection and those that have not, reinforcing its value as a predictor of the risk of dissection.

We provide tables that allow for the calculation of a Z-score, providing physicians with an objective assessment of where their patient lies compared to the expected values.

Furthermore, we propose a model for predicting type A aortic dissection risk at the time of the exam, though our model is not to extrapolate the risk over longer periods of time.

Future risk models may need to incorporate a combination of morphologic factors such as aortic length, along with demographic and potentially genetic factors, to improve dissection risk prediction accuracy.

## 6 Compliance with Ethical Standards

1. Guarantor:

The scientific guarantor of this publication is Dr David Rotzinger.

2. Statistics and Biometry:

One of the authors has significant statistical expertise.

3. Methodology.

Methodology:

• retrospective.
• cross sectional study.
• performed at one institution

## Informed consent:

Written informed consent was waived by the Institutional Review Board.

## Ethical approval:

Institutional Review Board approval was obtained.

## CRediT authorship contribution statement

**Thomas Saliba:** Conceptualization, Data curation, Formal analysis, Investigation, Methodology, Resources, Software, Validation, Writing – original draft, Writing – review & editing. **Gabriella Giandotti Gomar:** Conceptualization, Data curation, Formal analysis, Investigation, Methodology, Validation, Writing – original draft, Writing – review & editing. **Olivier Cappeliez:** Conceptualization, Data curation, Formal analysis, Investigation, Methodology, Project administration, Supervision, Validation, Writing – original draft, Writing – review & editing. **Yasser Alemán-Gómez:** . **Guillaume Fahrni:** Conceptualization, Data curation, Formal analysis, Investigation, Methodology, Supervision, Validation, Visualization, Writing – original draft, Writing – review & editing. **David Rotzinger:** Conceptualization, Data curation, Formal analysis, Investigation, Methodology, Project administration, Supervision, Validation, Writing – original draft, Writing – review & editing.

## Funding

The authors state that this work has not received any funding.

## Declaration of competing interest

The authors declare that they have no known competing financial interests or personal relationships that could have appeared to influence the work reported in this paper.
